# Associations between vicarious racism and psychoactive substance use depend on strength of ethnic identity

**DOI:** 10.1038/s41598-024-67202-7

**Published:** 2024-07-26

**Authors:** Isabela Cruz-Vespa, Sarah J. Dembling, Benjamin H. Han, Tristen K. Inagaki

**Affiliations:** 1 Department of Psychology, San Diego State University, San Diego, USA; 2 UCSD Department of Medicine, San Diego, USA; 3 SDSU-UC San Diego Joint Doctoral Program, San Diego, USA

**Keywords:** Psychology, Risk factors

## Abstract

Racism is a pervasive threat to health with differential impact based on race and ethnicity. Considering the continued perpetration and visibility of racism online and in the news, vicarious racism, or “secondhand” racism when hearing about or witnessing racism being committed against members of one’s ethnic or racial group, is a particularly urgent threat in the context of such disparities and their subsequent health consequences. The current study examines if frequency of exposure to vicarious racism and the emotional impact of those experiences are linked to psychoactive substance use, and explores the role of ethnic identity in moderating these relationships. In a cross-sectional survey, 504 adult participants aged 18–78 (*M* age = 30.15, *SD* = 11.52, 52.6% female) identifying as Black/African American or Latine reported on their experiences with vicarious racism and alcohol, marijuana, and tobacco use over the past 30 days. Logistic regression was utilized to test hypotheses. Primary findings indicate that greater emotional impact of vicarious racism was associated with a 50% increase in odds of alcohol consumption and that ethnic identity moderated the association between vicarious racism and marijuana use. Greater emotional impact of vicarious racism was related to more marijuana use for those lower on ethnic identity, whereas there was no association for those higher on ethnic identity. Vicarious racism was not related to tobacco use. Results suggest that ethnic identity might be protective in the association of vicarious racism on substance use. Further research on this topic is needed as vicarious racism becomes an increasingly common experience among marginalized populations.

## Introduction

No one is exempt from the basic need for social acceptance, connection and belonging. Threats to social connection are associated with higher mortality risk, increased chance of chronic disease and mental illness, and greater vulnerability to substance abuse and relapse^1^. While research on the negative effects of poor social connection in general is well-established, our understanding of how certain race-related threats to social connection contribute to health disparities is still limited. Racism contributes to a life-long source of vigilance and social stress^2–7^. A thorough understanding of exactly how and to what extent different facets of racism affect health remains understudied. The current cross-sectional study takes a preliminary step at addressing this gap by examining the relationship between vicarious racism, or “secondhand” racism, and psychoactive substance use, a health-relevant behavior.

Vicarious racism is an increasingly relevant facet of racism. Vicarious racism occurs when hearing about or witnessing racism being committed against other members of one’s ethnic or racial group through mediums such as social media, in the news, or from other people^8,9^. That is, anyone who identifies with the victim(s) may then experience this racism vicariously as well. This could involve, for example, watching news reports of police brutality, witnessing prejudice against one’s friends, family, or neighbors, or being subjected to distressing rhetoric of political leaders on social media. Social identity theory can offer one approach for understanding how this facet of racism affects social connection: self-identification with a target of racism can, by extent, negatively affect the self-concept and self-esteem of witnesses, thereby contributing to heightened social stress^5,6, 10–14^. Similarly, the racism-related stress framework posits that an individual's racism-related distress and hypervigilance can spread to those connected with that individual as well^15^.

Marginalized groups such as Black and Latine populations experience racism disproportionately at systemic, institutional, and interpersonal levels^16–20^. Through causing ongoing social and psychological stress, experiences of racism are suggested to lead to harmful health-risk behaviors such as smoking, excessive alcohol consumption or illicit substance-use^21^. Indeed, research has found evidence of substance use as a coping mechanism against feelings of stress and resulting deficits in self-regulatory processes, effectively helping to elevate mood^22–27^. Social stressors in particular are commonly found to trigger substance-use, abuse and relapse^28^. For example, experiences of social exclusion, stigmatization, disconnection, and neglect have all been linked to heightened substance-use and vulnerability to substance use disorders^24,28–34^. Furthermore, when such stressors are experienced on a chronic basis, the likelihood of developing a substance-use disorder is amplified^33,35^.

While experiences of racism in general meet criteria for being both a social and chronic stressor, relatively little research has focused on specific facets of racism, like vicarious racism, in relation to psychoactive substance use. Existing studies using Black and Latine samples have found associations between self-reported discrimination and increased risk of substance-use among African Americans, US-born Latines and immigrant Latines^36–41^. Burgeoning research on the effects of vicarious racism on these populations has revealed a connection with poorer mental health, lower life satisfaction, greater disease activity and even likelihood of juvenile crime^8,42–48^. One novel experimental study found a significant relationship between vicarious discrimination exposure and higher alcohol craving among Black young adults^49^. Annual substance-use reports find that while both Black and Latine populations consistently use substances at a rate lower than the white population, since 2020 both of these marginalized groups have experienced a significant rise in drug overdose-related deaths with the most disproportionate rates found among the Black population^50^. As various substances are known to increase an individual’s risk of developing serious health conditions when misused, it becomes increasingly important to further examine the relationship between vicarious racism and the risk of problematic substance-use.

Research has previously found evidence of highly visible (i.e. nationally publicized) instances of racism being the trigger of population-level changes in stress-related health consequences among members of targeted groups as well^51,52^. For example, one of the largest immigration raids in U.S. history was followed with state-level increases in adverse birth outcomes for children of immigrant Latina mothers but not non-Latina mothers^52^. Another study found that African American participants who lived in states where police had killed an unarmed Black person in the last 3 months reported more poor mental health days^51^. Considering that racially-charged information can be publicized regularly online and on social media, the chance of experiencing vicarious racism becomes increasingly more common among marginalized individuals^53–55^. Accordingly, vicarious racism has the potential to influence negative coping behaviors such as substance-use among populations at large as a result^16,55, 56^. With this significance in mind, the current study expands on existing research by exploring the relationship between frequency of exposure to vicarious racism, the emotional (i.e., subjective, psychological) impact of vicarious racism, and psychoactive substance use (tobacco, marijuana, and alcohol) over the past 30 days.

To better understand who might be most impacted by vicarious racism, we also assessed the role of a possible protective factor: ethnic identity. Ethnic identity refers to the strength of a person’s sense of self-identification with and belonging to their ethnic in-group or culture, as well as the value they attach to their ethnicity^57,58^. Foundational research posited a theoretical model of ethnic identity development, suggesting that this process involves stages of both exploration of and commitment to one’s ethnic identity^57^. If vicarious racism acts as an ongoing threat to social connection and source of stress, ethnic identity might moderate this relationship. Research has previously studied the buffering potential of ethnic identity and certain racial identity dimensions against the impact of racism on mental health, physical health, and substance use^43,59–66^. To our knowledge, however, there is only one existing study on the relationship between vicarious racism and substance use; in a sample of black young adults, Desalu et al.^49^ found that strong positive beliefs regarding Black racial identity (private regard) were protective against alcohol craving after a mild vicarious discrimination experience. Meanwhile, racial centrality was found to be exacerbating in an extreme experience^38^. To expand upon these existing findings, the current study explores how the construct of ethnic identity relates to the emotional impact of vicarious racism among both Black and Latine groups.

Two possible hypotheses were considered: (1) that ethnic identity would buffer the impact of vicarious racism, or (2) that it would exacerbate it. The argument for the former primarily considers the positive aspects of in-group identification, namely the resulting sense of group-based social connection, support, belonging and shared resources that help buffer against threats facing members of a community^57,67–71^. In this way, strong ethnic identity could function as a counterbalance against the ongoing threats to social belonging and connection posed by vicarious racism, thus protecting against substance use, consistent with previous findings linking racism and substance use^61,62,64^. The latter hypothesis, on the other hand, suggests that those who more strongly identify with their ethnic in-group would experience greater vicarious pain from witnessing racism or racial-violence directed toward that group^72–74^. Thus, ethnic identity could exacerbate, rather than protect against, vicarious racism-related substance-use.

## Methods

### Participants

Participants were recruited from Prolific and the psychology subject pool (SONA), two different online data collection platforms. Inclusion criteria were age 18 or over, current residence in the United States, English literacy, and identification as Latine and/or Black or African American. Considering that such research on vicarious racism is still in its early stages, it was decided to limit the sample to the two largest ethnic minority groups in the United States (2020 U.S. Census). Furthermore, recent data from 2022 shows notably high rates of mental illness, substance use disorder, and overdose deaths in particular among these two ethnic groups compared to others^75^.

Those who participated via the Prolific platform received $3 (*n* = 374) and those who participated via SONA received one research credit in exchange for participation (*n* = 130). The survey was approved by the Institutional Review Board of San Diego State University, and performed in accordance with all university regulations and guidelines. All participants provided informed consent prior to beginning the survey as well as a debriefing at the end of the survey. Data was collected from November 29, 2022 through May 21, 2023.

A minimum sample size of 284 participants was determined a priori via a power analysis in G*Power (Version 3.1;^76^) with an α of 0.05, power of 0.80, and a medium effect size (Cohen’s d between 0.3 and 0.5, based on the desired effect size) for the primary associations. The stopping rule for data collection, however, was to cease when we reached 500 participants in order to guard against data loss due to pre-determined exclusionary criteria (e.g., low quality responses, ignoring inclusion criteria). Thus, we excluded participants who did not identify as either Latine or Black/African American (*n* = 25), those who failed an attention check embedded within the survey, or those who completed the 20-min survey in under 4 min (*n* = 41). This resulted in an analytic sample size of 504: 212 Non-Latine Black, 280 Latine, and 12 Latine-Black/African American participants aged 18–78 (*M age* = 30.15, *SD* = 11.52, 52.6% female). See Table 1 below for additional demographic information. Analyses were not preregistered, but data needed to replicate results is publicly available (see Data Availability Statement).

**Table 1:** Sociodemographic characteristics of participants.

Baseline characteristic	Full sample	
	*n*	%
Sex assigned at birth^a^		
Male	239	47.4
Female	265	52.6
Race and ethnicity		
Non-Latine Black/African American	212	42.1
Latine	280	55.6
Latine-Black/African American	12	2.4
Marital status		
Never married	377	75
Divorced/separated/widowed	19	3.8
Married/cohabiting	107	21.3
Income		
Less than $20,000	85	17
$20,000 to $34,999	88	17.6
$35,000 to $49,999	72	14.4
$50,000 to $74,999	107	21.4
$75,000 to $99,999	58	11.6
Over $100,000	90	18
Highest educational level		
Less than high school	4	0.8
High school/GED	86	17.1
Some college	180	35.9
2-year College degree	47	9.4
4-year College degree	143	28.5
Master’s degree	32	6.4
Doctoral degree	4	0.8
Professional degree (JD, MD)	6	1.2
Employment		
Unemployed	149	29.6
Part-time	126	25
Full-time	201	40
Other	27	5.4

*N* = 504. Participants were on average 30.2 years old (*SD* = 11.5).

^a^Response options in this survey were Male or Female.

### Procedure

Participants were recruited for a 20-min online survey named “Health Behavior & Social Thoughts and Feelings.” Interested participants were redirected to Qualtrics where they completed measures of vicarious racism, ethnic identity, social connection, and substance use over the past 30 days. Additional health outcomes were collected as part of the cover story to measure general health and health behavior, but will be reported separately (self-rated health, sleep quality).

### Measures

#### *Vicarious racism*

Vicarious Racism was measured using an 8-item questionnaire adapted from a study that measured vicarious racism in Asian and Black Americans during the COVID-19 Pandemic^8^. In order to measure the frequency of exposure to vicarious racism experiences, participants rated how often they experienced vicarious racism over the past 30 days on a 0 (*never*) to 5 (*every day*) scale. Sample items included, “Other people of the same racial group experiencing racism in the news”, “Other people of the same racial group experiencing racism in public” and “Racist posts on social media”. Participants also reported the perceived emotional impact of their vicarious racism experiences (e.g., “Please rate how distressed or concerned you have been about the above experiences in the past 30 days.”) on a 4-point scale ranging from 0 (*never/not at all*) to 4 (*always/extremely*).

Previous studies tend to conflate the objective and subjective impact of vicarious racism^43,45^. It is possible, however, that the frequency with which one experiences vicarious racism and the subjective, or emotional, impact of such experiences show different associations with substance use. Thus, in the current study, we explored how frequency of vicarious racism and emotional impact separately relate to substance use. Frequency of vicarious racism experienced was scored by taking an average of the 6 frequency items (*M* = 2.6, *SD* = 0.833, *α* = 0.851). Emotional impact of racism experiences was scored by calculating the average of the 2 emotional impact items for each participant (*M* = 1.89, *SD* = 0.743, *α* = 0.803). Frequency of vicarious racism and emotional impact were related, but still separable (*r* = 0.604, *p* < 0.001, BCa 95% CI [0.523, 0.674]).

#### *Social connection*

The current theoretical perspective suggests vicarious racism represents a threat to social connection. To assess this possibility, feelings of social connection were measured with a two-item “state level feelings” social connection scale borrowed from previous research^77,78^ as well as the UCLA Loneliness Scale^79^. The two state-level feelings items were averaged such that higher scores indicate greater feelings of social connection (*M* = 2.8, *SD* = 0.997). The UCLA Loneliness Scale is a well-validated 20-item scale designed to measure one’s subjective feelings of loneliness as well as feelings of social isolation. Items were mistakenly collected on a 1–5, rather than 1–4 scale. In the current study, participants used the following scale: 1 = *Never*, 2 = *Rarely*, 3 = *Sometimes*, 4 = *Often*, 5 = *Always*. Higher scores indicate greater loneliness (M = 2.69, SD = 0.875; $\alpha$ = 0.961).

#### *Psychoactive substance use*

Psychoactive substance use was measured using The National Survey on Drug Use and Health (NSDUH), a questionnaire designed by the Substance Abuse and Mental Health Services Administration (SAMHSA), which asks about psychoactive substance (alcohol, marijuana, tobacco) and illicit substance use over the past 30 days (e.g., off-label prescription medication use, heroin). In the current study, we focused on questions regarding participants’ use of alcohol, marijuana, and tobacco because less than 2% of the sample endorsed illicit substance use and because psychoactive substances robustly predict health^80–82^. Participants were asked whether they had consumed any type of alcoholic beverage, marijuana or hashish, and tobacco (smoke, smokeless tobacco, cigars, smoked tobacco in a pipe, e-cigarettes or other vaping devices) over the past 30 days, including today. Answers to each of the three types of substances were coded as dichotomous outcomes (yes/no). Thus, the interpretation of the questions are, for example, “did you use [a substance] during the past 30 days—yes or no?”.

#### *Ethnic identity*

To measure ethnic identity, participants completed the Scale of Ethnic Experience^83^. Respondents are first asked to self-identify their ethnicity, and then respond to statements related to their experience of ethnicity on a 5-point scale (ranging from *strongly agree* to *strongly disagree*). Examination of its reliability and validity indicate that the SEE is a psychometrically sound measure of different facets of ethnicity that can be used across ethnic groups^83^. In the current study, the 12-item subscale of Ethnic Identity was used to measure both strength of ethnic in-group identification and value attached to one’s ethnicity, including items such as “I have a strong sense of myself as a member of my ethnic group”, “I believe that it is important to take part in holidays that celebrate my ethnic group”, and “My ethnic background plays a very small role in how I live my life.” Reverse scored items were re-coded so that higher scores meant stronger ethnic identity (*M* = 3.63, *SD* = 0.73; $\alpha$ = 0.881).

### Data analysis

To assess whether vicarious racism represents a threat to social connection, we ran correlations between frequency and emotional impact of vicarious racism and the social connection measures (i.e., ethnic identity, state-level feelings of social connection, loneliness). Correlations were run with Pearson correlations in SPSS. Significance for the correlations were determined based on a two-tailed *p*-value of 0.05 and a 95% confidence interval (CI) excluding 0.

Given that psychoactive substance use was collected as binary outcomes, associations between vicarious racism (frequency and emotional impact, separately) and substance use (alcohol, marijuana, and tobacco) over the previous 30 days were evaluated with logistic regression in STATA (v. 13). Additional logistic regression models were run controlling for frequency when testing emotional impact models, and vice versa. Data were evaluated for potential violations of model assumptions, but no violations were identified. The data met model assumptions of collinearity, (Tolerance < 0.800, variance inflation factor (VIF) < 4.00), independent errors (Durbin-Watson value = 2.020), and the assumption that the data did not contain influential cases that could influence the model (Cook’s Distance M = 0.002).

Current hypotheses also suggest that the association between vicarious racism and substance use may depend on the strength of ethnic identity. Therefore, additional logistic regression models investigated the moderating role of ethnic identity. After centering vicarious racism and ethnic identity, the two predictors and their interaction were entered into a logistic regression. For significant interactions, we ran follow-up, conditional logistic regressions examining the relationship between vicarious racism and substance use for individuals with “high” ethnic identity (scores above the mean) and “low” ethnic identity (scores below the mean). That is, separate logistic regressions were run for individuals with ethnic identity scores above and below the mean ethnic identification of the sample. Significance for logistic regressions were determined based on a two-tailed *p*-value of 0.05 and a 95% CI excluding 1.

Finally, results could be driven by other demographic factors. Indeed, age, sex, marital status, and indicators of socio-economic status (education level and annual household income) are related to substance use^8^. Therefore, these covariates were included in each logistic regression. Given that the data was collected on two separate platforms (SONA and Prolific), participant pool was also included as a covariate.

## Results

Consistent with the notion that vicarious racism represents a threat to social connectedness, both the emotional impact and frequency of vicarious racism were negatively correlated with social connectedness. Thus, greater emotional impact was associated with lower feelings of social connection (*r* = − 0.124, *p* = 0.005, BCa 95% CI [− 0.214, − 0.037]) and higher feelings of loneliness (*r* = 0.092, *p* = 0.038, BCa 95% CI [0.000, 0.190]). Higher frequency of vicarious racism was, likewise, associated with lower feelings of social connection (*r* = − 0.099, *p* = 0.027, BCa 95% CI [− 0.199, − 0.015]), but not with loneliness (*r* = 0.082, *p* = 0.067, BCa 95% CI [− 0.002, 0.181]). Interestingly, greater emotional impact of and exposure to vicarious racism was associated with stronger ethnic identity (*r*_*emotional impact*_ = 0.184, *p* < 0.001, BCa 95% CI [0.076, 0.275]; *r*_*frequency*_ = 0.234, *p* < 0.001, BCa 95% CI [0.145, 0.322]).

### Relationships between vicarious racism and psychoactive substance use

Primary hypotheses were that both the frequency and self-reported emotional impact of vicarious racism would be associated with more psychoactive substance use. We evaluated such associations separately for alcohol consumption, marijuana use, and tobacco use over the past 30 days.

Frequency of vicarious racism was not related to alcohol use. However, there was an association between emotional impact of vicarious racism and alcohol use, whereby an increase in emotional impact was associated with increased odds of alcohol use (Table 2). Specifically, for every point increase in the perceived emotional impact of vicarious racism, there was a 50% increase in odds of drinking alcohol. There were no associations between frequency or emotional impact of vicarious racism and marijuana or tobacco use over the past 30 days (Table 2).

**Table 2:** Relationship between vicarious racism (frequency or emotional impact) and substance use (alcohol, marijuana, or tobacco) over past 30 days. In each logistic regression model, the following variables were controlled for: age, sex, marital status, education level, annual income, and participant pool. Frequency of Vicarious Racism and Emotional Impact of Vicarious Racism variables were centered.

Vicarious racism	Psychoactive substance	Odds ratio	SE	z	*P* >|z|
Frequency	Alcohol	0.98	0.11	− 0.20	0.844
Emotional impact		1.50	0.20	3.07	0.002**
Frequency	Marijuana	1.08	0.14	0.56	0.573
Emotional impact		1.29	0.19	1.67	0.094
Frequency	Tobacco	0.96	0.12	− 0.29	0.768
Emotional impact		1.30	0.18	1.86	0.063

### Role of ethnic identity

To investigate the possible moderating role of ethnic identity on the link between vicarious racism and substance use, interactions between ethnic identity and vicarious racism (frequency and emotional impact, separately) were examined.

**Figure 1:**
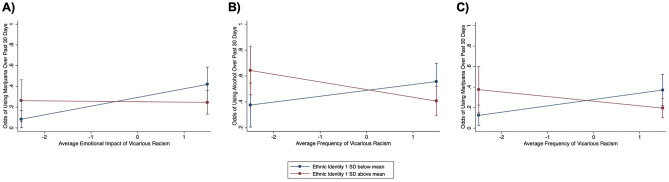
Moderation by ethnic identity. Simple slopes stratified by ethnic in-group identification for the relationship between vicarious racism and substance use with 95% confidence intervals. Blue lines represent “low” ethnic identity group with ethnic identity scores 1 standard deviation below the mean. Red lines represent “high” ethnic identity group with scores 1 standard deviation above the mean. Figures are for visualization purposes only; conditional regression analyses were conducted with “low” ethnic identity group defined as scores above the mean, and “high” ethnic identity group defined as scores above the mean. (**a**) There was a significant negative relationship between emotional impact of vicarious racism and marijuana use for the “low” ethnic identity group, but no relationship between the two variables for the “high” ethnic identity group. (**b**) Although there was an interaction between frequency of vicarious racism and ethnic identity for alcohol use and (**c**) marijuana, there were no significant relationships for either the “low” or “high” ethnic identity groups.

A significant interaction was found between ethnic identity and frequency of vicarious racism with alcohol use as a dependent variable, but not between ethnic identity and emotional impact of vicarious racism (Table 3, Fig. 1). To interpret the significant interaction, subsequent simple slope analyses were performed stratified by ethnic identity score. There was, however, no association between frequency of vicarious racism and alcohol use for individuals with low or high ethnic identity (Table 4). Although there were no significant associations for the low or high groups, there were differences in the direction of effects, whereby those with lower scores (below the mean) had a trending increase in odds of using alcohol with increases in exposure to vicarious racism and those with higher scores (above the mean) had a trending decrease in odds of using alcohol with increased vicarious racism.

In the models with marijuana use as a dependent variable, there was a significant interaction between ethnic identity and frequency of vicarious racism, as well as between ethnic identity and emotional impact of vicarious racism (Table 3, Fig. 1). Those with lower scores (below the mean) had trending increased odds of using marijuana with increases in exposure to vicarious racism and those with higher ethnic identity scores (above the mean) showed the opposite. However, neither simple effect was statistically significant (Table 4). Regarding the relationship between emotional impact of vicarious racism and marijuana use, increased emotional impact was related to increased marijuana use only for those low on ethnic identity. There were no associations between the two variables for those with high ethnic identity scores (Table 4).

Lastly, there were no significant interaction effects between ethnic identity and frequency or emotional impact of vicarious racism for tobacco use.

**Table 3:** Moderation by ethnic identity. Logistic regression results of interactions between vicarious racism (frequency or emotional impact) and ethnic identity. Each logistic regression model included vicarious racism (frequency or emotional impact), interaction between vicarious racism variable and ethnic identity, and the following covariates: age, sex, marital status, education level, annual income, and participant pool.

Vicarious racism	Psychoactive substance	Odds ratio	SE	z	*P* >|z|
Frequency	Alcohol	0.73	0.11	− 2.09	0.037*
Emotional impact		0.87	0.14	− 0.87	0.384
Frequency	Marijuana	0.66	0.12	− 2.27	0.023*
Emotional impact		0.68	0.13	− 1.98	0.048*
Frequency	Tobacco	0.90	0.14	− 0.70	0.486
Emotional impact		0.92	0.15	− 0.49	0.667

**Table 4:** Conditional logistic regressions for high ethnic identity and low ethnic identity. Results from conditional logistic regressions for high ethnic identity (self-reported ethnic identity above the mean; *n* = 268) and low ethnic identity (self-reported ethnic identity below the mean; *n* = 220) following significant interactions between vicarious racism and ethnic identity scores. The following covariates were included in each logistic regression: age, sex, marital status, education level, annual income, and participant pool.

Vicarious racism	Ethnic identity	Psychoactive substance	Odds ratio	SE	z	*P* >|z|
Frequency	High	Alcohol	0.74	0.12	− 1.82	0.068
	Low		1.23	0.21	1.21	0.225
Frequency	High	Marijuana	0.75	0.14	− 1.48	0.138
	Low		1.40	0.29	1.64	0.101
Emotional impact	High		0.83	0.18	− 0.85	0.394
	Low		1.86	0.45	2.55	0.011*

### Relationships between vicarious racism and psychoactive substance use, controlling for vicarious racism frequency and emotional impact

To understand how frequency and emotional impact of vicarious racism may relate to psychoactive substance use without the influence of one another, we ran additional logistic models that included both frequency and emotional impact of vicarious racism as independent variables. Therefore, we could examine the relationship between vicarious racism frequency and substance use while controlling for emotional impact of vicarious racism, and vice versa.

This altered the results in a few ways. First, when controlling for emotional impact of vicarious racism, frequency of vicarious racism was related to decreased, rather than increased, alcohol use (*p* = 0.009, 95% CI [0.50, 0.91], OR = 0.68).The positive relationship between emotional impact of vicarious racism and alcohol use became stronger when controlling for frequency (*p* < 0.001, 95% CI [1.41, 2.78], OR = 1.98).

There were still no statistically significant relationships between vicarious racism and marijuana use (frequency, controlling for emotional impact: *p* = 0.592, 95% CI [0.67, 1.26], OR = 0.92; emotional impact, controlling for frequency: *p* = 0.093, 95% CI [0.95, 1.95], OR = 1.36). Additionally, there remained no relationship between frequency of vicarious racism and tobacco use when controlling for emotional impact (*p* = 0.083, 95% CI [0.56, 1.04], OR = 0.76).

However, when controlling for frequency of vicarious racism, increased emotional impact of vicarious racism was associated with increased tobacco use (*p* = 0.012, 95% CI [1.10, 2.19], OR = 1.55). In particular, these results indicated that for every point increase in the perceived emotional impact of vicarious racism, there was a 55% increase in odds of using tobacco.

## Discussion

Threats to social connection put people at risk for health-risk behaviors such as psychoactive substance-use. Vicarious racism, or “secondhand” racism, is one understudied social stressor which disproportionately and chronically impacts marginalized groups, and thus may contribute to health disparities. Our cross-sectional study takes a step towards addressing this gap in the literature by showing that vicarious racism, particularly the emotional impact of vicarious racism, is linked to more alcohol consumption and tobacco use (when adjusting for the frequency of exposure to vicarious racism).

As previous studies have rarely differentiated between different components of racism when examining its impact on health, our study went beyond the current literature by showing that the perceived emotional impact of vicarious racism experiences may play a distinct role in how vicarious racism contributes to health disparities. Our findings reveal that the emotional impact of vicarious racism, but not exposure to it (i.e., frequency), is positively related to alcohol use. Emotional impact of vicarious racism was also related to more tobacco use, above and beyond the frequency of exposure to vicarious racism. Unexpectedly, our findings also indicated a negative relationship between frequency of vicarious racism and alcohol use when controlling for emotional impact of vicarious racism. Possible explanations for these findings are that the emotional impact of experience drives psychoactive substance use rather than objective measures of how many times one is exposed, and focusing on the frequency of vicarious racism alone could be hiding the emotional effects of vicarious racism. Although future experimental research is needed to test this possibility, it may be the case that experiences of vicarious racism can be harmful if they cause significant emotional distress for the individual, regardless of frequency of exposure. On the other hand, if one does not perceive experiences of vicarious racism to be emotionally distressing, it may not have a harmful impact.

Future research may further examine individual nuances of this perceived emotional impact by considering the subjective, emotional and stress-related mechanisms through which racism has been theorized to influence health. Among them, previous literature has proposed heightened perceptions of injustice and loss of social status, traumatic stress, increased fear of personal victimization, increased demand on psychological coping resources, diminished trust in social institutions, anger, and communal bereavement^51,84^. A better understanding of these mechanisms will aid researchers and community health workers to bring awareness to how the emotional impact of vicarious racism ultimately influences health as well as to design effective interventions and resources for marginalized communities, even while they are not able to necessarily reduce exposure to vicarious racism.

Our findings also support the hypothesis that stronger ethnic identity may buffer the impact of vicarious racism; those reporting lower ethnic identity tended to show positive associations between vicarious racism (both the emotional impact and frequency of experiencing vicarious racism) and alcohol and marijuana use whereas those higher in ethnic identity did not. This suggests that individuals who more strongly identify with their ethnic in-group may be less likely to turn to these substances when exposed to vicarious racism. In the U.S., marginalized individuals experience vicarious racism (as well as other facets of racism) chronically, and at higher levels than non-marginalized^17,85^. Such experiences may benefit from the sense of community support, social connection, social belonging, meaning, validation, historical awareness, shared resources and collective agency that strong ethnic identity can provide. As such, further research should work to improve our understanding of ethnic-identity as a potential form of protection from vicarious racism by examining if any aspects of ethnic identity have a stronger influence on substance use than others.

Of note is that the current study did not measure substance-use disorder or dependence, but rather general psychoactive substance-use, which does not necessarily imply problematic use. In fact, according to the Global Commission on Drug Policy, the most common pattern of drug use is episodic and non-problematic^86^. Our results echo those of previous studies on substance use related to distress; the most frequently reported substances used in our sample were alcohol, marijuana, and tobacco, rather than any illicit drugs measured. Nevertheless, alcohol is one of the substances with the highest likelihood of developing into dependence and has among the highest morbidity rates^87,88^. Moreover, this is one of the most easily accessible substances for both youth and adults alike^89,90^. Problematic use of alcohol can result in conditions such as heart and liver damage, stroke, cancer, and diabetes, thus placing a tremendous burden both on the individual as well as on society^91^.

## Limitations and future directions

The findings of this study are subject to various limitations. First, this study was cross-sectional, meaning that causal inferences cannot be made. Future research on coping behaviors related to vicarious racism may benefit from designing longitudinal, experimental studies utilizing a manipulation in order to simulate vicarious racism. The current study also relied on self-report for all measures. Although many of these measures were well-validated and widely-used, future research may attempt to measure real-time exposure to vicarious racism and intentions to use substances in daily life.

Furthermore, as the online-survey designed for this study was administered in English, non-English-speaking people living in the U.S. who would otherwise have been eligible for the study were not able to participate. The 2019 U.S. Census found that the percentage of individuals who spoke a language other than English at home had grown by over 50% since 2000, with nearly 20% speaking English either “not well” or “not at all”^92^. Previous studies have found that immigrants may be particularly vulnerable to racial discrimination and racism-related substance-use, and that they simultaneously experience multiple barriers to accessing health care^41,93–99^. Relatedly, the current study did not consider acculturation level, a factor that may impact vulnerability to racism-related stress. There is research to suggest that lower acculturation levels buffer this stress, and that ethnic identity may have greater protective potential for foreign-born Latines than U.S.-born Latines^100–104^. Future research should therefore measure and account for the acculturation level of participants. Questionnaires utilized to measure racism-related constructs and health-risk behaviors, such as a vicarious racism scale, should also become more widely validated in commonly spoken non-English languages in the U.S., such as Spanish and Chinese.

Furthermore, the current study did not distinguish between the different demographic subgroups, which are likely to have varying experiences of vicarious racism and ethnic identity. Previous research has found variation in how subgroups of both Black-American and Latine-American populations experience direct racism^103,105–108^. For example, in a study measuring self-reported racism exposure among US-born vs. foreign-born Black pregnant women, US-born subjects reported significantly more exposure to racial discrimination than foreign-born^105^. It was noted that the longer a subject had resided in the US, the more exposure to racism they reported. In regards to ethnic identity, one study found that while stronger ethnic identity was associated with less discrimination-related distress among foreign-born Latine adults, the opposite was apparent among U.S.-born Latine adults^103^. In regard to vicarious racism, however, studies have yet to explore associations by these subgroups in Black Americans. Research on vicarious racism among Latine samples is even more limited^46–48^. Future studies would thus do well to explore nuances in the experience of vicarious racism and ethnic identity between subgroups such as foreign-born and U.S.-born, first and second generation, age, and country of origin.

Additionally, while the current study sample included Latine and Black groups, these are only two of several ethnic groups in America subject to vicarious racism. Very little research thus far has considered the effects of vicarious racism or ethnic identity on the health of other ethnic minority groups, including growing immigrant and asylee populations^8,109–112^. We hope that future research will take the next step along our line of inquiry by incorporating other targeted ethnic groups, such as Asian-American, Indigenous, and Middle Eastern, into more study research samples.

Two limitations specifically relate to the construction of the measures used. While previous studies have often recommended the use of multiple, connected scales to capture individual components of racial and ethnic identity, the current study instead utilized a composite ethnic identity variable in order to create a more concise survey^59,113^. Future studies could expand on these findings by including a more extensive measure. Additionally, there are no vicarious racism scales yet which have been properly validated for use; the version used in the current study is adapted from a previous study on vicarious racism but has not yet been through rigorous psychometric testing. This scale measured a composite variable as well; new versions could be devised which better show the differential impact between sources of vicarious racism (e.g., social media vs. news on the television). Research has previously found higher rates of vicarious racism to be reported among certain contexts, such as those related to law enforcement^44,51^. Future research should thus work on validating a vicarious racism questionnaire in order to allow researchers to properly collect much-needed data on different aspects of this construct, especially as vicarious racism becomes more pervasive in the modern era.

Another limitation regards some of the study results. Although we found that the link between frequency of vicarious racism and substance use (alcohol and marijuana) depends on ethnic identity, it is important to note that the simple effects at high and low levels of ethnic identity were not significant. Future research should examine this interaction with larger sample sizes, include a more precise measure of alcohol and marijuana use (e.g., with a daily diary), and/or consider a manipulation of ethnic identity rather than a self-report measure.

## Conclusion

The current findings are a preliminary step towards understanding and preventing the harmful impact of vicarious racism, a pervasive threat to social connection. This is important as the possible mediums for vicarious racism expand in modern society; constant media coverage, television and radio news streaming, smartphones and social media platforms all play roles in enabling unprecedented levels of exposure. Despite vicarious racism acting as a constant message of social devaluation and exclusion, marginalized populations often face the expectation of just continuing on with daily life. On the other hand, the resulting increase in awareness of racism-related events has also led to racial justice-focused mass movements promoting civic-engagement, accountability measures, and centering the voices of marginalized groups. Taking this into consideration, it is crucial that future work promotes anti-racism and social change as a preventive public health strategy, in addition to designing interventions to help buffer racism’s immediate health impacts. Based on findings of the current study, interventions might consider integrating the significance of ethnic identity as a potential target for the prevention and reduction of psychoactive substance use among Black and Latine Americans. This could involve the further funding and cultivation of environments that encourage exploration of one’s ethnicity as well as commitment to ethnic or racial pride even in the face of vicarious racism experiences.

## Data Availability

Data and full code for analyses are available on the Open Science Framework: https://osf.io/arhfj/
